# Disseminated *Mycobacterium tuberculosis* in Imported Sooty Mangabey, Thailand

**DOI:** 10.3201/eid2103.131785

**Published:** 2015-03

**Authors:** Sawang Kesdangsakonwut, Angkana Sommanustweechai, Angkana Chaiprasert

**Affiliations:** Chulalongkorn University, Bangkok, Thailand (S. Kesdangsakonwut);; Ministry of Public Health, Muang Nonthaburi, Thailand (A. Sommanusweechai);; Mahidol University, Bangkok (A. Chaiprasert)

**Keywords:** Tuberculosis and other mycobacteria, sooty mangabey, Thailand, zoonoses, bacteria

**To the Editor:** Tuberculosis caused by bacteria of the *Mycobacterium tuberculosis* complex affects humans and various species of captive and free-living wildlife (*1*). In addition, *M. tuberculosis* has been used experimentally in many different species of Old World monkeys as part of the attempt to establish a suitable model for human tuberculosis (*2*). We report a case of disseminated tuberculosis caused by *M. tuberculosis* Spoligotype International Type (SIT) 52 in a recently imported sooty mangabey (*Cercocebus atys*) from South Africa to Thailand.

A juvenile male sooty mangabey was imported from South Africa to Thailand in September 2009. Within 1 week, while in quarantine, convulsion and salivation developed in the mangabey, and it died suddenly. This animal, along with another mangabey and 4 mustached guenons (*Cercopithecus cephus*), was imported from its native Africa to Thailand for the pet trade. Complete histories of the second mangabey and the mustached guenons were not available.

A complete necropsy of the dead sooty mangabey was conducted, and full histopathologic and microbiological analysis was performed. At necropsy, the mangabey was emaciated, with no subcutaneous and abdominal fat tissues. Disseminated granulomas (up to 2 cm) were observed throughout the carcass, including the lungs, liver, spleen, kidneys, multiple lymph nodes (hilar, mediastinal, mesenteric, splenic, hepatic, renal, and pancreatic), and the ileum. The lung was also multifocally adhered to the thoracic wall and pleural diaphragm.

Histologically, the granulomas in all tissues examined demonstrated similar histopathologic features, characterized by a central core of caseous necrosis and surrounded by an unorganized rim of mixed inflammatory cells, including neutrophils, lymphocytes, plasma cells, and epithelioid macrophages. Numerous acid-fast bacilli were present in the cytoplasm of the epithelioid macrophages and in the necrotic area of all tissues. Acid-fast bacilli were isolated and classified as *M. tuberculosis* on the basis of 1-tube multiplex PCR (*3*) and sequencing of 16S rRNA gene results. Spoligotyping revealed that the *M. tuberculosis* isolate belonged to SIT 52.

The international wildlife trade had been reported to be a major source of imported zoonoses, particularly tuberculosis, in nonhuman primates (*4*–*8*). In the mangabey reported here, fulminant tuberculosis was diagnosed within 1 week after it arrived in Thailand, during the 21-day quarantine period. The granulomas were morphologically similar to the histopathologic description of tuberculosis lesions of experimentally infected cynomolgus macaques (*Macaca fascicularis*), which demonstrated lesions as early as 3 weeks after infection, with a gradual increase in severity (*2*). Previously, East African–Indian lineage (*9*) and Beijing spoligotype (SIT 1) accounted for most *M. tuberculosis* isolates in Thailand (*10*). In nonhuman primates in Thailand, *M. tuberculosis* complex had been detected at rates of up to 50% (5 positive samples from 10 test samples) by PCR from buccal swabs in long-tailed macaque (*Macaca fascicularis*) (*1*). *M. tuberculosis* belonging to SIT 52 observed in this case has been primarily isolated from countries in Africa (*9*). Only 1 case of *M. tuberculosis* belonging to SIT 52 that caused tuberculous meningitis was reported in a human in Thailand (*10*), but that case was not related to the case reported here. Our finding of a relatively novel spoligotype of *M. tuberculosis* in an animal destined for the pet trade underscores the need for intensive testing of and extended quarantine for all imported nonhuman primates to prevent the spread of newly isolated *M. tuberculosis* (*4*,*7*,*8*).
